# Measuring the Invisible: The Sequences Causal of Genome Size Differences in Eyebrights (*Euphrasia*) Revealed by k-mers

**DOI:** 10.3389/fpls.2022.818410

**Published:** 2022-07-29

**Authors:** Hannes Becher, Jacob Sampson, Alex D. Twyford

**Affiliations:** ^1^School of Biological Sciences, Institute of Evolutionary Biology, University of Edinburgh, Edinburgh, United Kingdom; ^2^Royal Botanic Garden Edinburgh, Edinburgh, United Kingdom

**Keywords:** k-mers, genome size, *Euphrasia*, structural variation, transposable element, copy number, satellite DNA

## Abstract

Genome size variation within plant taxa is due to presence/absence variation, which may affect low-copy sequences or genomic repeats of various frequency classes. However, identifying the sequences underpinning genome size variation is challenging because genome assemblies commonly contain collapsed representations of repetitive sequences and because genome skimming studies by design miss low-copy number sequences. Here, we take a novel approach based on k-mers, short sub-sequences of equal length *k*, generated from whole-genome sequencing data of diploid eyebrights (*Euphrasia*), a group of plants that have considerable genome size variation within a ploidy level. We compare k-mer inventories within and between closely related species, and quantify the contribution of different copy number classes to genome size differences. We further match high-copy number k-mers to specific repeat types as retrieved from the RepeatExplorer2 pipeline. We find genome size differences of up to 230Mbp, equivalent to more than 20% genome size variation. The largest contributions to these differences come from rDNA sequences, a 145-nt genomic satellite and a repeat associated with an Angela transposable element. We also find size differences in the low-copy number class (copy number ≤ 10×) of up to 27 Mbp, possibly indicating differences in gene space between our samples. We demonstrate that it is possible to pinpoint the sequences causing genome size variation within species without the use of a reference genome. Such sequences can serve as targets for future cytogenetic studies. We also show that studies of genome size variation should go beyond repeats if they aim to characterise the full range of genomic variants. To allow future work with other taxonomic groups, we share our k-mer analysis pipeline, which is straightforward to run, relying largely on standard GNU command line tools.

## Introduction

Over the past century, cytogenetics researchers have uncovered various genomic features such as repetitive neocentromers ‘knobs’ (e.g., Creighton and McClintock, 1931), heterochromatin (Heitz, 1928) and B chromosomes (Jones, 1995 and references therein). These are all associated with structural genomic variation and genomic repeats, which, in turn, contribute to genome size variation. As recent and ongoing advances in DNA sequencing technology have revolutionised the community’s ability to characterise genetic variation at the sequence level, it is now possible to study, at unprecedented detail, the sequences underpinning genome size variation within and between closely related species.

Genome size is a trait directly affected by structural genomic variation. For example, a deletion of a part of the genome results in a smaller genome size. Because of the ubiquity of structural genomic variation in populations, including ploidy differences, supernumerary chromosomes, segmental duplications and other ‘indels’, the assumption of intraspecific genome size variation is a plausible null hypothesis. However, the magnitude of this variation and whether it can be detected by methods such as microdensitometry or flow cytometry has been subject to debate, and some older reports have been refuted (Greilhuber, 2005; Suda and Leitch, 2010). Nevertheless, flow cytometry studies following best practices and using internal reference standards have revealed genome size variation within numerous species including bottle gourds (Achigan-Dako et al., 2008), grasses (Šmarda et al., 2010; Díez et al., 2013), clubmosses (Hanušová et al., 2014), pinks (Terlević et al., 2022) and metazoans (Blommaert, 2020).

Genome size shows a staggering 2,400-fold variation across species of embryophyte plants (Pellicer et al., 2018). Within this range, a larger genome size is generally associated with higher proportions of genomic repeats as detected by low-pass sequencing studies, although genome repetitiveness was found to be somewhat lower in the species with the largest genomes (Novák et al., 2020a). The repeats accounting for most of the DNA in plant genomes can be classified into two categories: interspersed and tandem (satellite) repeats (Heslop-Harrison and Schwarzacher, 2011), both of which may affect genome evolution in characteristic ways. Interspersed repeats correspond to transposable elements (transposons) which due to their copy-and-paste (or cut-and-paste) nature can insert themselves into distant parts of the genome. Crossing over between such elements can lead to chromosomal rearrangements, associated with DNA loss or duplication, reviewed in Charlesworth et al. (1994). Over evolutionary time, there may be bursts of transposon activity (e.g., Jiménez‐Ruiz et al., 2020) possibly triggered by ‘genomic shock’ (e.g., Petit et al., 2010), but short-term change of their copy numbers is usually low. Satellite repeats on the other hand consist of numerous copies arranged in a head-to-tail fashion. Although some satellite repeats are extremely conserved (Abad et al., 1992), they are generally known for rapid changes in copy number and sequence identity between species (Tek et al., 2005; Kovarik et al., 2008; Koukalova et al., 2010; Ambrozová et al., 2011; Becher et al., 2014; Ávila Robledillo et al., 2020), within populations (Veltsos et al., 2009; Rabanal et al., 2017) and between the sub-genomes of allopolyploids (Heitkam et al., 2020). Satellite copy number has been shown to correlate with genome size, for instance in the case of rDNA arrays (Davison et al., 2007; Long et al., 2013), and in maize chromosomal knobs (Chia et al., 2012).

Despite the highly advanced state of DNA sequencing and the existence of genome assemblies for many species, it is still challenging to pinpoint the genomic sequences underlying intraspecific genome size variation. This is because structural variation commonly includes genomic repeats, which are often misassembled or missing even in high-quality genome assemblies (Schmid et al., 2018; Subirana and Messeguer, 2018). Alternative approaches based on low-pass sequencing by design miss low-copy number sequences. In this article, we will demonstrate that comparing the k-mer inventories of two individuals allows one to pinpoint in a straightforward way which sequences and genomic copy number classes contribute to genome size differences.

**Figure 1:**
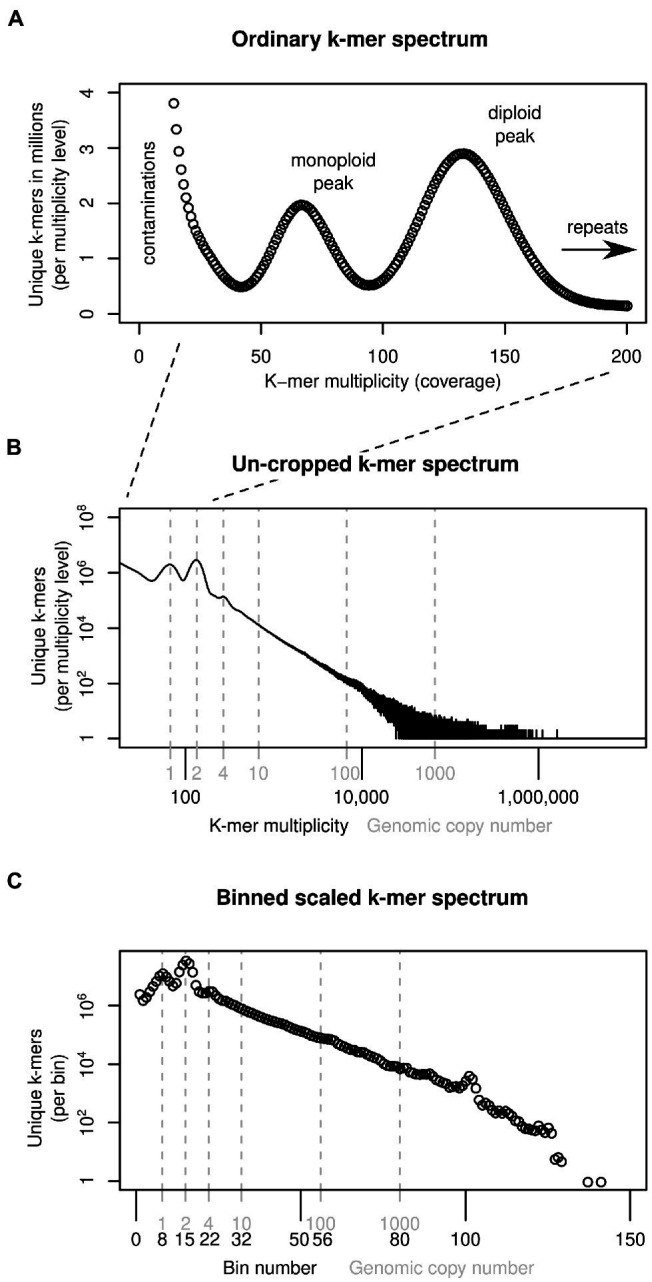
Ways of depicting individual-sample k-mer data sets. Panel **(A)** shows a k-mer spectrum with linear axes and the multiplicity (*x*-axis) cropped at 200, excluding k-mers present in genomic repeats. To represent all sample k-mers, the axes may be scaled logarithmically as in **(B)**. To compare samples, the multiplicity values can be scaled and binned **(C)**. See main text for more detail.

K-mers are short sub-sequences of equal length, *k*, that can be generated from DNA sequencing reads. The approach we introduce here builds on widely used k-mer spectra, which represent how many unique k-mers there are (*y*-axis) for each observation frequency level (multiplicity, *x*-axis). For instance, in an example k-mer spectrum of a diploid in Figure 1A, there are two peaks. The monoploid peak contains sequences present only in one genome (heterozygous sites), and the second peak contains sequences identical between the two genome copies (homozygous sites). Repeats are not covered by this plot, which tend to be cropped to an arbitrary multiplicity level (here 200), just above the diploid level. To represent all k-mers in a genome, an ‘un-cropped’ k-mer spectrum may be plotted with logarithmic axes, as in Figure 1B. Here, the *x*-axis is labelled with both multiplicity values (black) and the corresponding genomic copy number (grey). The ratio between multiplicity and genomic copy number depends on each individual sample’s sequencing depth. If two samples are to be compared, the multiplicity values must be rescaled to be comparable, a natural scale being the genomic copy number. To reduce the range of copy number values that are compared, the data may be binned, as shown in Figure 1C, which reduces the number of comparison points to approximately 130 bins (from several 100,000 shown in Figure 1B). Because binning is carried out after scaling, a bin number corresponds to the same genomic copy number (range) in all samples.

Several hypotheses exist as to the sequences causing genome size differences in closely related species and populations. Here, we investigate three hypotheses, which are not mutually exclusive. (1) Genome size differences may be due to satellite repeats. Satellite repeats are known for their propensity for rapid copy number change as mentioned above and are thus natural ‘suspects’ for causing genome size differences. (2) Differences may be caused by sequences ‘across the board’—all kinds of sequences proportional to their genomic copy number. Recombination between distant repeat elements may cause the duplication, loss or translocation of larger chromosome fragments resulting in copy number changes of numerous sequences ‘across the board’ (Vitales et al., 2020). (3) Size differences may be due to low-copy number sequences. Numerous pangenome studies (e.g., Cao et al., 2011; Gan et al., 2011; Gordon et al., 2017; Hübner et al., 2019) have found variation in low-copy number sequences between individuals of the same or closely related species.

In this study, we use high-coverage (≥20×) shotgun data to investigate the sequences underlying genome size variation in diploid British eyebrights (*Euphrasia* L.), in which we have previously uncovered considerable intraspecific genome size variation (Becher et al., 2021). These diploids form a complex of hybridising taxa, which are not distinguishable by DNA barcoding (Wang et al., 2018) albeit there is some congruence between morphology and patterns of variation of amplified-fragment length polymorphisms (French et al., 2008). We intentionally avoid using assembly-based approaches, which we have previously used to investigate species differences (Becher et al., 2020). Instead, we compare genome size and genome composition by means of k-mers, allowing us to investigate the whole spectrum of genomic repetitiveness.

## Materials and Methods

### The Study System

Eyebrights (*Euphrasia* L., Orobanchaceae) are a genus of facultative hemiparasitic plants with a largely bipolar distribution (Gussarova et al., 2008). All British species are summer annuals, and are either diploids or tetraploids. The diploids—on which we focus here—tend to have large showy flowers and are mixed-mating or outbreeding (French et al., 2005). They carry an indumentum of long glandular hairs and are largely restricted to England and Wales (Metherell and Rumsey, 2018). We have previously identified 1.2-fold genome size variation among 40 diploid individuals (Becher et al., 2021).

### Sampling and Sequencing

Our k-mer analyses require high-coverage sequencing for multiple individuals and species. We collected three additional samples to complement previously generated sequence data available for four *Euphrasia* individuals (see Table 1). Diploid samples were collected in the field and stored in silica gel for desiccation (see Table 1 for details). We used the UK grid reference finder^1^ to convert sample coordinates to degrees and to compute a geographic distance matrix between all sample locations. In total, our sampling covered a geographic range of 570 km (between samples Vi-Ro). Where we included multiple individuals per species, each individual came from a different population, with the closest pair of samples being Ri1 and Ri2 which were collected 2.5 km apart (Table 2).

**Table 1:** Sample information for diploid *Euphrasia* species used in this study.

ID	Species	Read length	Cov^*^	NCBI ID	% het^*^	GS (Mbp)^*^	GS Diff^§^	Platform^†^	Lat/Long	1C (pg)^‡^	This study
An1	*E. anglica*	2 × 250 bp	54	SAMN14582932	0.13	999.98	NA	6	50.514/−4.113	0.51	
An2	*E. anglica*	2 × 150 bp	28.5	SAMN23180913	0.85	989.23	−10.75	6	51.845/−4.145	0.51	X
Vi	*E. vigursii*	2 × 150 bp	42.4	SAMN14582918	0.14	1055.93	55.95	X	50.24/−5.381	0.54	
Ro	*E. rostkoviana*	2 × 250 bp	67.4	SAMN14582916	1.13	1227.92	227.94	6	55.058/−2.504	0.63	
Ri1	*E. rivularis*	2 × 150 bp	35	SAMN14582917	0.23	1126.64	126.66	X	54.534/−3.192	0.58	
Ri2	*E. rivularis*	2 × 150 bp	25.5	SAMN23180914	1.41	1096.44	96.46	6	54.513/−3.203	0.56	X
Ri3	*E. rivularis*	2 × 150 bp	20.8	SAMN23180915	1.41	1104.84	104.87	6	53.082/−4.084	0.56	X

* Cov - multiplicity of the monoploid k-mer peak, % het - heterozygosity in %, GS - genome size per diploid genome in Mbp, each as inferred using Tetmer.

† Sequencing platform: X - Illumina HiSeq X, 6 - Illumina NovaSeq 6000.

§ Difference in Mbp to reference individual An1.

‡ Converted from k-mer estimate to pg following Doležel et al. (2003).

**Table 2:** Pairwise genome size differences (lower triangle) and geographic distances (upper triangle) between sampling sites.

		An1	An2	Ri1	Ri2	Ri3	Ro	Vi	
Diploid GS diff (Mbp)	**An1**		148.06	451.36	448.94	285.62	516.74	94.91	Distance (km)
	**An2**	10.75		305.66	303.22	137.63	373.40	198.25	
	**Ri1**	126.66	137.40		2.45	171.72	73.09	499.95	
	**Ri2**	96.46	107.20	30.20		169.27	75.40	497.50	
	**Ri3**	104.87	115.61	21.79	8.41		242.67	328.41	
	**Ro**	227.94	238.68	101.28	131.48	123.07		569.66	
	**Vi**	55.95	66.70	70.71	40.51	48.92	171.99		

We extracted DNA of the newly collected samples using the DNeasy Plant Mini Kit (Qiagen, Manchester, United Kingdom) according to the manufacturer’s instructions. Truseq Nano libraries, incorporating eight PCR cycles, were constructed by Edinburgh Genomics, who generated 150-bp paired-end reads on an Illumina NovaSeq 6000 instrument.

### Handling k-mer Data

#### Generating k-mer Data Sets and Estimating Genome Sizes

Subsequent to read trimming and filtering with fastp v0.22.0 (Chen et al., 2018) with automatic detection of sequencing adapters in paired-end mode (flag ‘--detect_adapter_for_pe’), we generated k-mer databases for each sample using the software KMC3 (Kokot et al., 2017). Throughout this project, we used 21-mers (k-mers of length 21).

In order to remove k-mers of organellar origin, we generated crude *de novo* assemblies of one plastid and one mitochondrial genome using GetOrganelle (Jin et al., 2020) and used these to produce organellar k-mer databases. KMC3’s default settings are designed for sequencing datasets (not assemblies) and therefore exclude k-mers with a multiplicity one, which would likely to be due to sequencing errors. At a k-mer size of 21, many k-mers observed in an organellar genome assembly will be observed only once. To make sure all assembly k-mers were included in the organellar databases, we ran KMC3 with parameter ‘-ci1’. We then used KMC3 to exclude organellar k-mers from each sample database.

For each sample, we generated three uncropped k-mer spectra (i.e., with the upper multiplicity limit set to 150,000,000, far higher than observed in our data): one for the full (but trimmed and filtered) read data, one with plastid k-mers removed and one both with plastid and mitochondrial k-mers removed. We profiled these datasets using GenomeScope2/Smudgeplot (Ranallo-Benavidez et al., 2020), and Tetmer (Becher et al., 2020).

From these un-cropped, cleaned k-mer spectra, we estimated the diploid genome size for each individual as follows. We discarded the portion of each spectrum with multiplicity less than half the individual’s monoploid peak multiplicity—largely the contamination peak. For the remaining data, we multiplied the multiplicity and count values. We then took the sum of these products, and divided by the monoploid multiplicity. For conversion to pg. (picogram, 1 × 10^−12^ grams), we followed Doležel et al. (2003).

#### Scaling and Binning

To compare the number of k-mers within each frequency (multiplicity) class between samples, we had to scale the multiplicity values of our datasets. We determined for each sample the monoploid (‘haploid’) k-mer multiplicity using the Tetmer app^2^ (Becher et al., 2020), and down-scaled the multiplicity values of each k-mer spectrum accordingly so that the resulting spectra had their monoploid peaks at 1 (see Figures 1B,C). The scaled multiplicity values corresponded to the genome-wide copy number of each k-mer (plus some statistical sampling error caused by shotgun sequencing). However, because each sample had a different monoploid multiplicity, the resulting fraction-valued scaled multiplicity values differed between samples. To compare samples, we binned these scaled multiplicities. Throughout this article, we use the terms scaled (binned) multiplicity and (genomic) copy number interchangeably.

To easily analyse the full range of genomic copy numbers, we decided to use unequal bins, increasing in size in an exponential fashion. We discarded all scaled multiplicities equal to or less than 0.5 because these were likely due to contaminants. We then generated bins (copy number classes) with upper limits 10% larger than their lower limits {(0.5, 0.55], (0.55, 0.605], …, (20.57,22.63], …}. The total number of bins used may differ between samples with the highest bin number corresponding to the highest-copy number k-mer in any dataset. We also generated alphabetically sorted k-mer dumps with KAT3. These are two-column text files of k-mers and their respective multiplicity in a dataset.

#### Comparing k-mer Data Sets

Using *E. anglica* (An1) as the reference individual and building on data scaled and binned as described above, we generated two types of sample comparisons: k-mer difference graphs and joint k-mer spectra.

##### Difference Graphs

**Figure 2:**
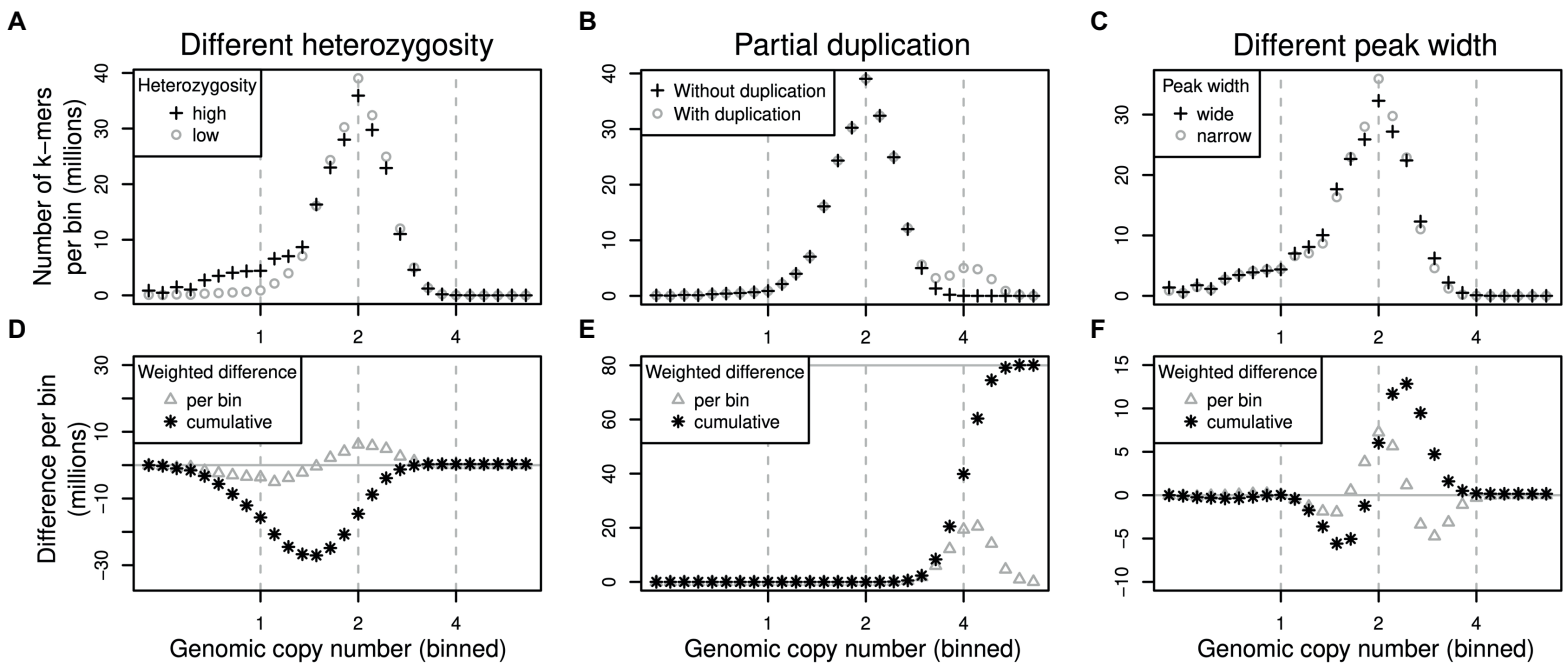
Schematic of pairs of (binned) k-mer spectra (top row) and their corresponding spectrum difference graphs (bottom row). Three different scenarios are shown in columns: (1) two samples of identical genome size with different heterozygosity levels **(A,D)**, (2) two samples where one contains some additional, duplicated sequence **(B,E)** and (3) two samples with identical sequences but whose k-mer spectra have different peak widths **(C,F)**. Refer to main text for detailed explanations.

To quantify how much the k-mer differences in each copy number bin contribute to the overall genome size difference between two samples, the per-bin differences are multiplied by the expected copy number of k-mers in each bin. The total genome size difference between two samples can then be obtained by summing over all per-bin products (analogous to computing the genome size from a k-mer spectrum). We generated k-mer difference graphs that indicate the contribution of each copy number bin to the overall genome size difference. This type of comparison is ignorant of sequence identity. Difference graphs can also be plotted in a cumulative way with the graph’s ‘slope’, indicating the contribution to the genome size difference of any one specific bin. Figure 2 illustrates for three scenarios how these graphs correspond to the underlying data (here focussing on low-copy number regions).

The scenarios shown in Figure 2 are: (1) If one sample has a higher heterozygosity than the other (Figure 2A), but the samples have identical genome sizes, then the high-heterozygosity sample (crosses) will show a higher 1x peak but a somewhat lower 2x peak than the other sample (circles). The difference graph for this scenario (Figure 2D) will show two peaks in opposite directions at 1x and 2x (Figure 2D, triangles). The cumulative difference graph (Figure 2D, stars) will cross the 1x line with a steep slope indicating a high difference in copy number for 1x k-mers. This is compensated by a steep slope in the opposite direction for 2x k-mers causing a net genome size difference of 0 (vertical grey line). (2) If two samples are identical except for some sequence which is absent in one sample but present at copy number 4 in the other, then one k-mer spectrum will have an additional peak at 4x (Figure 2B, circles). The corresponding difference graph will show a peak at 4x (Figure 2E, triangles) and the cumulative difference graph will show a steep slope at 4x leading to a non-zero overall difference (Figure 2E, stars). (3) Different k-mer datasets may have different peak widths even when generated from the same biological sample (technical replicates) depending on the method of library preparation and the sequencing platform chosen. Wider peaks tend to be shallower (Figure 2C, crosses) than narrow ones (Figure 2C, circles). This effect may not be obvious in a binned k-mer spectrum, but it does affect difference graphs (Figure 2F). While not causing an overall genome size difference, the resulting cumulative difference graph shows a downtick followed by a steep increase crossing *x* = 2 followed by another decrease back to 0 (Figure 2F, stars). This pattern would be inverted if the samples were swapped.

##### Joint k-mer Spectra

A joint k-mer spectrum of two samples is a matrix that shows how many k-mers from two datasets were observed at each combination of multiplicities. In this way, a joint spectrum is aware of sequence identity. The k-mer difference graph of two samples contains only a subset of the information of the joint k-mer spectrum. We generated binned joint k-mer spectra by matching up pairs of k-mer dumps (analogous to database joins on the k-mer column). We then scaled and binned the counts in these joins, which reduced the number of count levels from millions to approximately 150 bins. Finally, we counted the number of times that each combination of two bin values occurred, resulting in a three-column table (count, bin number in the reference and bin number in the other sample), and we converted this table into a matrix, the binned joint k-mer spectrum. These joint spectra can be visualised as heatmap plots, making it possible to show copy number differences between two whole genomes in a single plot.

#### Contribution of Different Repeat Types

To associate any genomic copy number differences identified using k-mers with specific repeat types, we used the RepeatExplorer2 (RE) output of a previous study (Becher et al., 2021), in which we had carried out an analysis of low-pass sequencing data of several diploid and tetraploid British eyebrights. We selected the first 50 repeat superclusters and concatenated, per supercluster, all contributing reads. We then used the program UniqueKMERS (Chen et al., 2021) to extract from each concatenated sequence those k-mers that were unique to the corresponding supercluster, and we turned the concatenated sequences into 50 k-mer databases with KMC3. We used these databases to extract from each of the seven high-coverage datasets 50 subsets of repeat k-mers. Finally, we generated joint k-mer spectra for each of these subsets and the corresponding data from reference individual *E. anglica* (An1).

## Results

### Genome Profiling

Our genome profiling revealed k-mer patterns typical for diploid genomes in all our samples (Table 1). The monoploid k-mer coverage of our datasets ranged from 20.8 in *Euphrasia rivularis* (Ri3) to 67.4 in *E. rostkoviana* (Ro). Per-nucleotide heterozygosity as estimated by Tetmer ranged from 0.13% in *E. anglica* (An1) to 1.41% in *E. rivularis* (Ri2 and Ri3). Samples with very low heterozygosity (such as An1, Vi and Ri1), containing very few heterozygous k-mer pairs, did not have a noticeable ‘AB’ smudge in Smudgeplot analyses (Supplementary Material). In consequence, Smudgeplot incorrectly suggested these samples were tetraploids, while proposing all samples with higher levels of heterozygosity were diploids. Spectrum peak widths (bias parameters) varied considerably between individuals from 0.9 in Ri2 to 2.4 in Vi.

By comparing uncropped k-mer spectra before and after removal of organelle sequences, we could infer the distributions of organellar k-mers (Supplementary Material). These had one peak for mitochondrial k-mers (green) but two for plastid k-mers (red). The high multiplicity of these peaks indicated the high copy number of organellar genomes compared to nuclear. The second peak in the plastid-derived k-mers was located at approximately twice the copy number of the first peak and presumably corresponded to the two copies of the plastid inverted repeat region. Using un-cropped spectra with organellar k-mers removed, we estimated the ‘2C’ genome sizes of our samples to range more than 1.2-fold from 989 Mbp in *E. anglica* (An2) to 1,227 Mbp in *E. rostkoviana* (Ro). For comparison, without organellar DNA removed, these estimates were 3.8 to 7.2% higher. The individual genome size estimates showed a clear partitioning by species, with species identity accounting for 98.6% of the variation (ANOVA, *F*_3,3_ = 72.43, *p* = 0.0027). Repeating the ANOVA on permuted versions of the dataset showed that this *p*-value and proportion of variance explained are unlikely to occur by chance (one-sided 95% confidence interval).

### Difference Graphs

**Figure 3:**
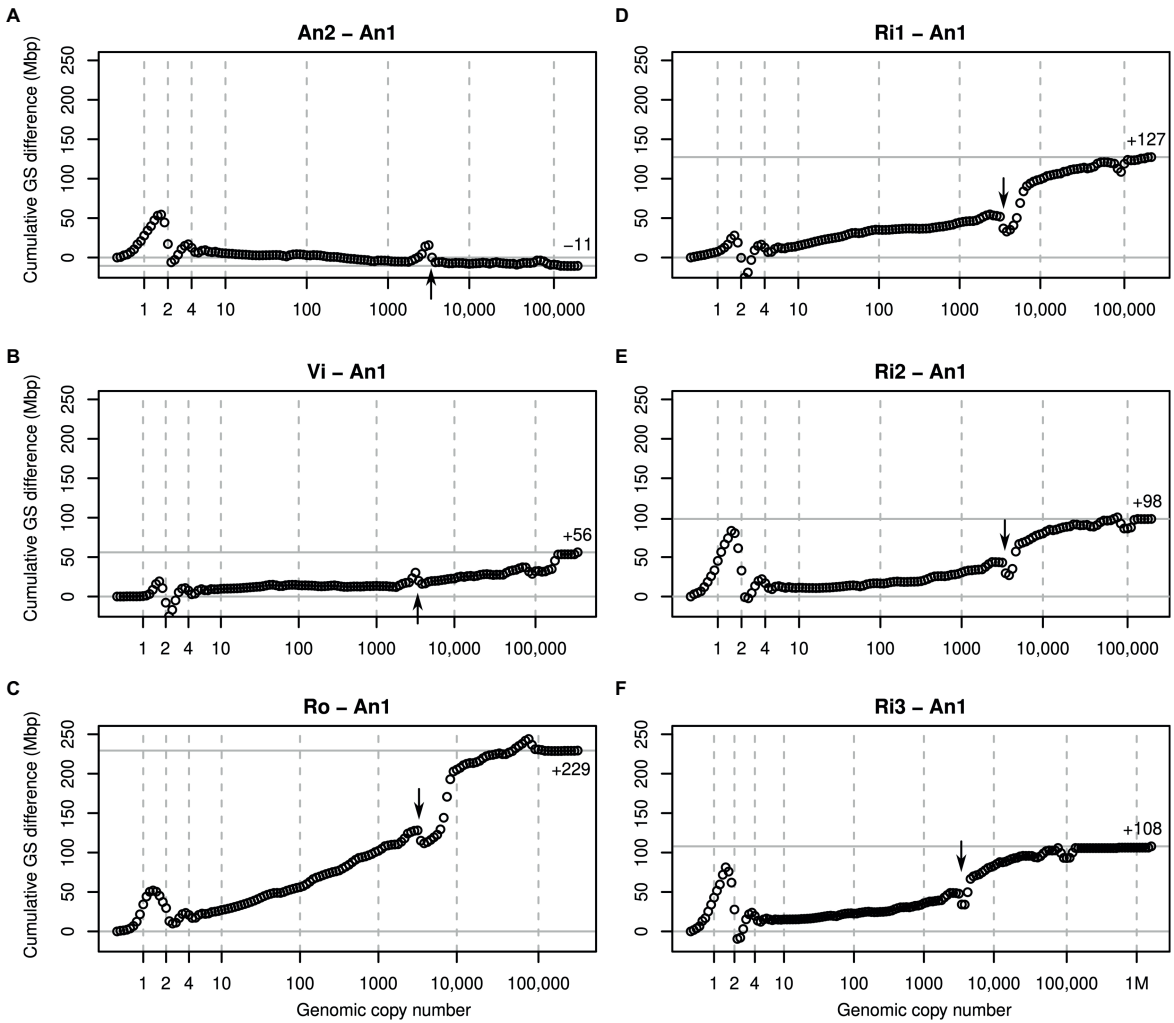
Cumulative k-mer difference graphs detailing the contributions to genome size differences of genome fractions ordered by increasing repetitiveness for six samples of diploid *Euphrasia* compared to diploid *Euphrasia anglica* (An1). The numbers on the x-axes indicate the genomic copy number bins with 1, 2 and 4 representing haploid, diploid, and ‘duplicated’ sequences. The genome size differences are shown on the y-axes, scaled identically for all graphs. The total genome size difference between the two samples in each graph is indicated at the right-hand side of each plot and by a horizontal grey line. The arrows indicate a ‘gap’ caused by copy number variation of a repeat present in approximately 3,000 copies in the reference individual. The panels show comparisons six different individuals to the reference: **(A)** *E. anglica*, **(B)** *E. vigursii*, **(C)** *E. rostkoviana*, **(D-F)** *E. rivularis*.

We generated cumulative k-mer difference graphs for all samples compared to reference individual An1 (Figure 3). These graphs indicated very similar magnitudes of genome size differences to those obtained from un-binned, un-cropped spectra (Table 2). This suggests that binning, despite reducing the information content of our data, did not bias our inferences.

Comparisons of low-heterozygosity *E. vigursii* (Vi, Figure 3B) and *E. rivularis* (Ri1, Figure 3D) to the low-heterozygosity reference individual of *E. anglica* (An1) did not reveal large differences in heterozygous k-mer counts (which, by definition, have monoploid copy number in diploids), and the curves were flat at *x* = 1. All other samples had higher levels of heterozygosity than the reference individual causing a positive difference in k-mer count leading to a positive slope where the data line intersects with the vertical line at *x* = 1 (Figures 3A,C,E,F). Because these are cumulative plots, peak differences correspond to slopes (as shown in Figure 2, lower row). All samples showed negative slopes where the data line crossed the diploid (*x* = 2) and duplication (*x* = 4) copy number bins. By the time the cumulated data series reached *x* = 10, there were no strong up or downticks, and all samples had a somewhat higher number of k-mers than the reference individual.

Across the rest of the copy number range, all plots changed largely gradually and nearly monotonically. That is, across bins, k-mer count differences tended to have the same sign. An obvious exception from this was a more or less prominent ‘gap’ in all plots near *x* = 3,000 (see arrows in Figure 3). This pattern is consistent with a repeat of about 3,000 copies in the reference sample (An1) and with different copy numbers in the other samples. If a sample contained a lower copy number of this repeat than the reference, then it showed an excess of repeat k-mers at a lower copy number followed by a drop at *x* = 3,000 as seen in An2 (Figure 3A) and Vi (Figure 3B). If, however, a sample contained more copies of this repeat than the reference, then the plots showed a deficiency at *x* = 3,000 and a subsequent excess as seen in all other samples (Figures 3C–F). A similar but less pronounced pattern was seen at approximately *x* = 100,000 in most plots.

### Joint k-mer Spectra and Repeat Types

To assess the contribution to genome size differences of individual genomic repeats, we matched up k-mers from our samples with k-mers specific to the 50 largest repeat superclusters identified in a previous study in *Euphrasia*. Collectively, these accounted for approximately 50% of the *Euphrasia* genomes, and the smallest of these superclusters corresponded to a genome proportion of approximately 0.06%. Across samples, the variation in k-mers associated with these repeats accounted for 57 to 78% of the genome size differences observed. Because we only used k-mers unique to individual superclusters, this is likely an underestimate. The only exception was the difference between the *E. anglica* individuals (An2-An1) where the difference in repeat-associated k-mers exceeded the overall genome size difference by 9%. The fact that the An2 genome was larger than predicted based on repeat k-mers suggests that it contained an excess of lower-copy number k-mers compared to the reference individual An1.

**Figure 4:**
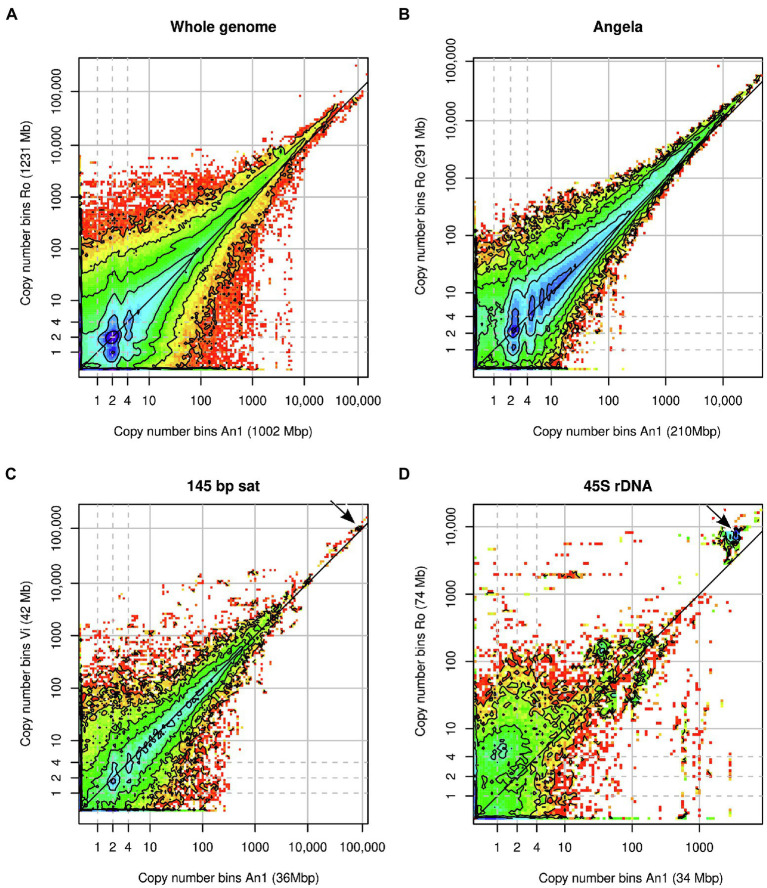
Heatmaps of binned joint k-mer spectra in comparative analyses of *Euphrasia* genomic sequences. **(A)** shows the comparison of whole genomes. The other panels show k-mers associated with specific genomic repeats: **(B)** Angela LTR retrotransposon, **(C)** 145-bp satellite, **(D)** 45S rDNA. Copy number bins of the reference individual are shown on the *x*-axis. The axis labels show in parentheses the contribution of the k-mer fraction depicted to each individual’s overall genome size. The dashed grey lines indicate haploid, diploid and ‘duplicated’ copy numbers. The dark grey diagonal line in each plot indicates the zone where copy numbers are equal between the samples. The arrows in panels **(C,D)** indicate k-mer clusters responsible for the gap patterns in Figure 3B and Figure 3C.

Heatmaps of joint k-mer spectra (Figure 4) revealed in more detail how k-mer fractions associated with genomic repeats that differed between samples. Figure 4A shows the comparison of all genomic k-mers between Ro and An1. The high heterozygosity of sample Ro showed as dark blue colour at *y* = 1 with the highest counts at *y* = 1 and *x* = 2, indicating that most k-mers found at heterozygous sites in Ro are present in two copies in An1. There is no corresponding high density of k-mers at *x* = 1 and *y* = 2, which agrees with our previous finding of An1 being a low-heterozygosity individual. In the higher-copy number (>1,000) regions of the plot, high k-mer densities are found above the diagonal line, indicating higher repeat copy numbers in Ro than An1.

The repeats with the most variable contribution to genome sizes were superclusters 1, 4 and 2, which correspond to a Copia transposable element of the family Angela, the 45S rDNA and a 145-bp satellite repeat, respectively. By plotting the joint k-mer spectra for individual repeat types, we could match the gap patterns seen in the cumulative difference graphs (Figure 3). The patterns at 100,000x correspond to the 145 bp-satellite (Figure 4C) and the one at 3000x to the 45S rDNA (Figure 4D). While the latter two panels contain numerous lower-copy number k-mers, the genome size differences caused by these repeats are accounted for by compact clusters of high-copy number satellite k-mers located off the diagonal line (indicated by arrows). The Angela-associated k-mers showed a more diffuse pattern, with k-mers of multiplicity >1,000 showing a higher abundance in Ro than in An1 (off-diagonal tiles in Figure 4B).

### The Importance of Different Copy Number Ranges

**Figure 5:**
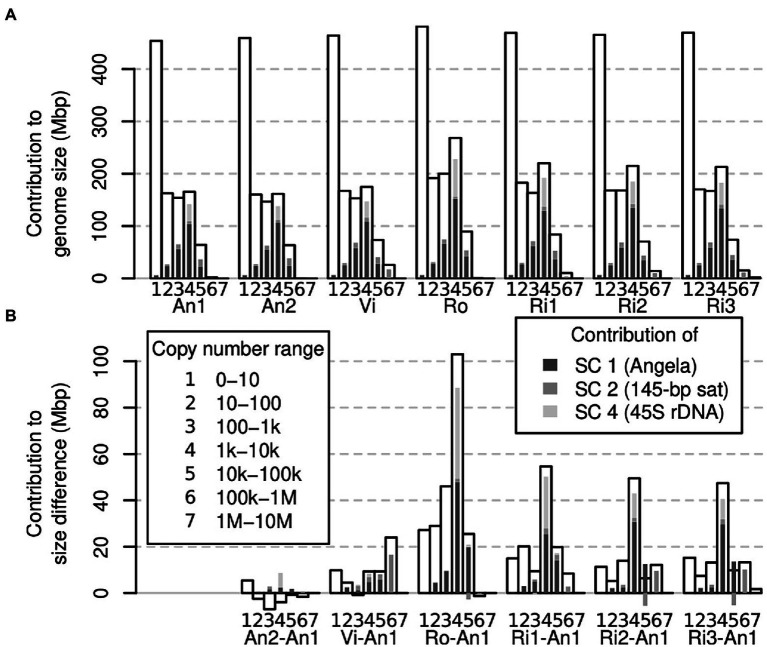
Contribution of different genomic fractions to overall genome size **(A)** and genome size differences **(B)** in *Euphrasia* genomic data. The contributions of repeat superclusters 1, 2 and 4 are indicated in shades of grey as indicated in the legend, which applies for both panels.

To assess which genomic copy number ranges contribute to the overall genome size of an individual, we binned our k-mer spectra even more coarsely and compared across all samples. Figure 5A shows that for all individuals, the copy number range 0–10 was the single largest class. However, taken together, the other copy number ranges contained more k-mers. The three copy number ranges, 10–100, 100–1,000 and 1,000–10,000, contained similar amounts of k-mers, each usually less than half the amount of the 0–10 range. All higher copy number ranges were smaller. For comparison, we highlighted the contributions to each copy number range of the three largest repeat superclusters 1, 2 and 4 (supercluster 3 corresponded to plastid DNA, which we had removed from our data sets).

While a large part of our samples’ genomes were accounted for by low-copy number sequences (Figure 5A), we found that the range contributing most to genome size differences was that of 1,000–10,000 copies. Most of the differences in this range were driven by sample differences in Angela and 45S rDNA k-mers (Figure 5B).

## Discussion

In this study, we developed an approach for studying differences in genomic composition within and between closely related species, using British eyebrights (*Euphrasia*) as a test case. Rather than using genome assemblies or low-pass sequencing data, we compared the contents of genomes by means of a k-mer approach using high coverage data, which allowed us to inspect the whole range of genomic copy number classes. We found that all copy number classes contributed to genome size differences with large contributions from a few individual repeats notably including an Angela transposable element. Below, we compare our approach to other existing methods, we critically assess its robustness, and then we turn to what we have learned about eyebright genome evolution.

### Comparison to Other Approaches

The content of two or more genomes may be compared in several ways. Perhaps, the most obvious is to use whole-genome alignments, which has been practiced for more than two decades (e.g., Chinwalla et al., 2002; Armstrong et al., 2020). Such studies have revealed how genome structure changes over time, for instance following hybridisation and whole-genome duplication (Chalhoub et al., 2014). However, most genome assemblies are still not complete, lacking faithful representation of their repetitive sequences. Such sequences are commonly represented in collapsed form or are missing (remaining ‘invisible’) due to the problem of assembling repeats comprising monomers longer than the sequencing read length. Also, genome assemblies usually attempt to represent in one sequence the two (or more) genome copies present in an individual, which may differ in size. Current assembly-based approaches are thus unlikely to comprehensively answer the question of genome size differences. Nonetheless, pangenome studies, which compare multiple genomes of closely related species or individuals, have ubiquitously shown that there is structural variation in populations and between closely related species including presence/absence variation of low-copy number sequences (Golicz et al., 2016; Gordon et al., 2017; Hübner et al., 2019).

An alternative approach, focusing only on high-copy number sequences, is the analysis of low-pass genome sequencing data (‘genome skimming’, Straub et al., 2012). Because most eukaryote genomes contain more repeats than low-copy number sequences, genome skimming studies can reveal sequences with major contributions to genome size differences. A popular method is RepeatExplorer2 (Novák et al., 2010, 2013, 2020b), which takes a set of short low-pass shotgun sequencing reads, constructs clusters of similar reads and assembles from these repeat consensus sequences. The repeat clusters are then annotated using a curated database. RepeatExplorer2 can also analyse multi-individual datasets to compare the genome composition of multiple samples, usually of different species. Such studies have convincingly shown differences between species in repeat patterns without the need for a genome assembly, and plausibly linked these to genome size differences (Ågren et al., 2015; Macas et al., 2015). However, genome skimming studies by design miss single- and low-copy number regions, which also contribute to genome size differences between individuals (Lower et al., 2017).

The approach we chose here may be categorised as a ‘genome profiling’ method, where the properties of genomes are investigated by means of k-mers using moderately high-coverage sequencing data, but in the absence of a genome assembly. Other genome profiling methods have been developed to assess assembly completeness (KAT; Mapleson et al., 2016), sequence contamination and heterozygosity (GenomeScope; Vurture et al., 2017) and ploidy (Smudgeplot; Ranallo-Benavidez et al., 2020) and to estimate population parameters (Tetmer; Becher et al., 2020). Unlike these single-individual methods, we compared pairs of individuals, generating joint k-mer spectra—matrices that simultaneously show the copy number of k-mers in two individuals. K-mer multiplicities of individual samples tend to range from one to several millions. Squaring this number, a full joint k-mer spectrum would be too large to handle computationally. A key aspect of our approach was to bin multiplicity levels, reducing what would be huge un-cropped joint k-mer spectra to matrices of approximately 150 × 150 bins without losing relevant information. We used these binned joint spectra to compare copy number differences in genome sequences of any copy number, from heterozygous and homozygous single-copy regions (Figure 4A, blue areas) to satellite repeats (copy number > 100,000, Figure 4C).

Beyond comparisons of genome size and composition, our approach may also be used to assess how similar genomes are. This can be achieved by converting per-bin k-mer differences into Gower (or Manhattan) distances. When multiple samples are analysed, this approach can then be used to construct a distance matrix which in turn can be used to cluster samples or to generate a phylogeny similar to other alignment-free methods (Dodsworth et al., 2015; Ondov et al., 2016; Herklotz et al., 2021).

### Measuring Genome Size Differences With k-mers

Knowing about the shortcomings of genome assemblies, which tend to be smaller than genomes size estimates obtained by flow cytometry (Bennett et al., 2003), we utilised a k-mer approach. Despite this, we found our bioinformatic estimates of genome size were all lower (except for Ro, 1C = 0.63 pg) than 40 previous estimates for diploid *Euphrasia* species, obtained by flow cytometry (Becher et al., 2021). The lowest of these previous estimates was 1C = 0.6 pg. While possible, it seems unlikely that most of our samples truly contained less DNA than all samples analysed previously.

The discrepancy between expected and observed genome size values could not be due to sequence contamination with non-target DNA, which would have increased, not reduced our estimates. The fact that we removed organelle-derived k-mers from our datasets might have wrongly removed nuclear sequences of organelle origin such as NUMTs or NUPTs, which are known in the family Orobanchaceae (Cusimano and Wicke, 2016), thus biasing our estimates downward. However, these sequences usually account for a negligible amount of the nuclear genome (Hazkani-Covo et al., 2010; Lloyd et al., 2012). Another possibility is that our sequencing data did not contain a faithful representation of the genome contents of our samples due to some intrinsic bias in the library preparation or sequencing technology (Pfeiffer et al., 2018). It is also notable that different k-mer-based tools produce different genome size estimates, suggesting that some models are more accurate than others (Melsted and Halldórsson, 2014; Sarmashghi et al., 2021). Finally, there is also error associated with genome size estimates made with flow cytometry, most notably that certain dyes will bind to particular sequence motifs (Doležel et al., 1998), and that sizing is made indirectly relative to a reference standard (which is also subject to associated error). There are other examples where genome size estimates obtained from k-mer spectra are smaller than flow cytometry (Sun et al., 2018; Mgwatyu et al., 2020), suggesting that this may be a general issue worthy of future study.

### All Frequency Classes Contribute to Eyebright Genome Size Differences

It would seem plausible that low-copy sequences contribute more to genome size variability in species with small genomes. In contrast, genome size differences between large genomes may be driven mostly by differences in repeat abundances. Here, we found that all copy number classes contributed to genome size differences between our samples. Across most samples, different copy number fractions contributed similar amounts to the overall genome size difference except for the sequences in the copy number fraction 1,000–10,000 (Figure 5B), many of which were 45S rDNA and thus satellite sequences. We also detected a considerable contribution to genome size differences of repeat supercluster 2, which was associated with a 145-bp tandem repeat, possibly centromeric, in samples Vi, Ri2 and Ri3 (Figure 4B). These observations confirm our hypothesis (1) that satellites contribute in a major way to *Euphrasia* genome size differences.

While all copy number classes contributed to genome size differences, these contributions did not correlate well with the proportion they contributed to each genome (compare Figures 5A,B). For instance, low-copy number sequences (0 to 10 copies per genome) formed the largest class (> 400 Mbp) in all genomes. But, this class was proportionally underrepresented among the sequences that cause genome size differences. This shows that genome size differences are not a consequence of sequences across the board *per se*, and we refute our hypothesis (2). However, we cannot exclude the possibility that recombination between distant repeat copies led to copy number changes across numerous sequences. This is because different copy number fractions may not be distributed uniformly along *Euphrasia* chromosomes. For instance, studies on multiple species of angiosperms have revealed that genomic repeats and single-copy sequences tend to be located in different regions of the chromosomes (Barakat et al., 1998; Bertioli et al., 2019), while in bread wheat, gene density increases along chromosomes away from the centromeres (Akhunov et al., 2003). Although this pattern is not universal (Lang et al., 2018), if it was to hold in *Euphrasia*, structural variation caused by recombination between transposable elements might affect repeat sequences disproportionally more than low-copy number sequences.

Finally, all samples contained more low-copy DNA (copy number ≤ 10) than the reference individual *E. anglica* (An1), ranging from an additional 5 to 27 Mbp at the diploid level (Figures 3, 5B). Although this is modest compared to the overall genome size differences between samples, it shows that there is a considerable contribution to genome size differences from low-copy number sequences, which confirms our hypothesis (3). This finding also calls for a *Euphrasia* pangenome study to assess the differences in gene space between *Euphrasia* individuals, which we are currently working on.

### Genome Comparisons and Our Understanding of Diploid British *Euphrasia*

British *Euphrasia* have become known for their taxonomic complexity (*sensu* Ennos et al., 2005). While the diploids are largely morphologically distinct from one another (although numerous diploid hybrid combinations are known), they cannot be distinguished reliably by ITS or plastid barcoding (Wang et al., 2018), raising the question whether they are genetically distinct. Adding to this doubt, we have also recently uncovered considerable intra- and interspecific genome size variation within ploidy levels and showed that ‘population’ is a far better predictor of an individual’s genome size than ‘species’ (Becher et al., 2021). As such, our current working hypothesis has been that *Euphrasia* species may not show genome-wide differentiation, and, instead, species differences may be maintained by a few genomic regions under strong selection while the rest of the genome experiences homogenising gene flow.

These previous findings contrast with our results here, which indicated that genome size is predicted well by morphological species identity and that there are considerable copy number differences in Angela transposable elements between species. Transposable elements are generally thought to show lower rates of copy number change than other genomic repeats and they tend to be dispersed throughout genomes. Divergence in TE copy number might thus indicate genome-wide divergence between the diploid species of British *Euphrasia*, possibly resulting from a ‘genomic shock’ following hybridisation. This divergence may not show in the ITS sequences, which due to their repetitive nature tend to show a different turnover behaviour than other nuclear loci. Genetic divergence between species may also be missed when analysing plastid sequences, which tend to have lower substitution rates and effective population sizes (Ennos et al., 1999). Introgression (or ‘capture’) of plastid genomes (Percy et al., 2014; Liu et al., 2020) is another phenomenon that might conceal differentiation in the nuclear genomes. It is worth noting, however, that TEs and other repeats may accumulate in genomic regions of low recombination and may thus have a propensity to segregate in large blocks. Being mindful of this possibility and our limited sampling design, the species-specific genome size differences we revealed here may be seen as further evidence for diploid British *Euphrasia* being more distinct species than their tetraploid relatives (French et al., 2008).

## Data Availability Statement

The datasets generated for this study can be found in the sequence read archive. The sample identifiers are detailed in Table 1. The analysis code was deposited on GitHub https://github.com/hannesbecher/genome-size-variation.

## Author Contributions

HB and AT conceptualised the study. HB carried out the analyses and wrote the manuscript. JS developed the methodology and original code guided by HB and AT. AT secured funding and collected samples. All authors contributed to the article and approved the submitted version.

## Funding

This work was funded by NERC grants (NE/R010609/1; NE/L011336/1; NE/N006739/1) awarded to AT.

## Conflict of Interest

The authors declare that the research was conducted in the absence of any commercial or financial relationships that could be construed as a potential conflict of interest.

## Publisher’s Note

All claims expressed in this article are solely those of the authors and do not necessarily represent those of their affiliated organizations, or those of the publisher, the editors and the reviewers. Any product that may be evaluated in this article, or claim that may be made by its manufacturer, is not guaranteed or endorsed by the publisher.

## Footnotes

^1^ https://gridreferencefinder.com

^2^ https://github.com/hannesbecher/shiny-k-mers
