# Supplementary material for: Measuring the Invisible: The Sequences Causal of Genome Size Differences in Eyebrights (Euphrasia) Revealed by k-mers
Source: Front Plant Sci. 2022 Jul 29;13:818410. doi: 10.3389/fpls.2022.818410 (PMC9372453; doi:10.3389/fpls.2022.818410)
Supplement: Supplementary file 1 [file Data_Sheet_1.DOCX]

Supplementary Material

# Supplementary Text 1. Genome profiling

All k-mer analyses were carried out with *k*=21. The top two panels show a plot and fitted parameters generated with the Tetmer app (<https://github.com/hannesbecher/shiny-k-mers>, Becher et al., 2020 <https://doi.org/10.1016/j.xplc.2020.100105>). The bottom left panel shows the Smudge Plot. The bottom right panel shows the full k-mer spectrum with log axes. Mitochondrial and plastid k-mers are shown in green and red, respectively.

## An1 – *Euphrasia anglica*

Individual of the *E. anglica* assembly.

| 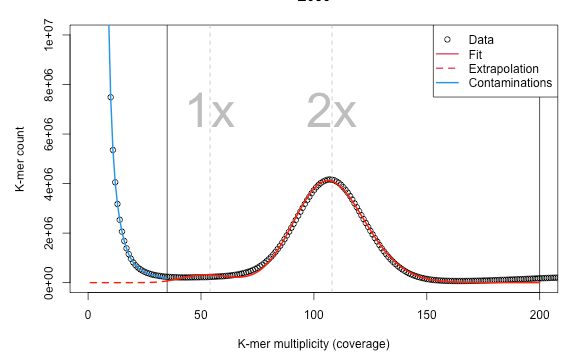 | DIPLOID MODEL, AUTO FITTED   k-mer length: 21   monoploid k-mer cov: 54   θ per k-mer: 0.027   θ per nucleotide: 0.00128   non-rep GS (Mbp): 165.5   bias (peak width): 1.3  STARTING RANGES (MIN MAX)   monoploid k-mer cov: 10 61   log10 θ per k-mer: -2 0.6   non-rep GS (Mbp): 1 2000   bias (peak width): 0.1 3   x range: 35 200 |
| --- | --- |
| 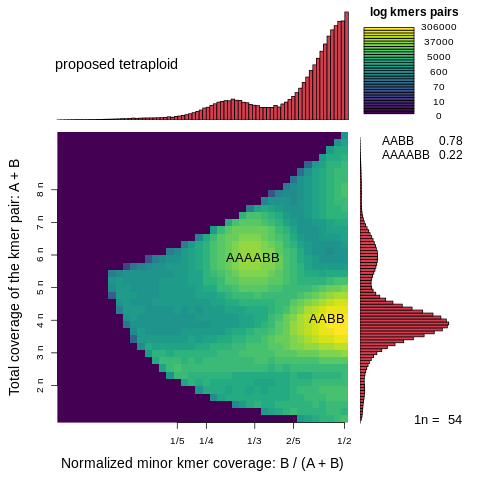 Smudgeplot wrongly proposes tetraploidy. The sample is actually diploid but with very low heterozygosity. The AABB peak corresponds to duplicated regions. | 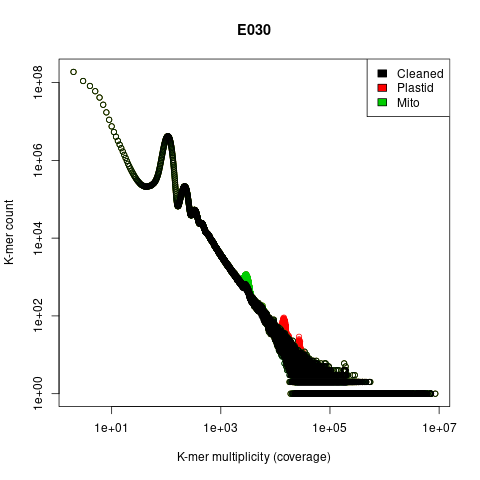 |

## An2 – *Euphrasia anglica*

| 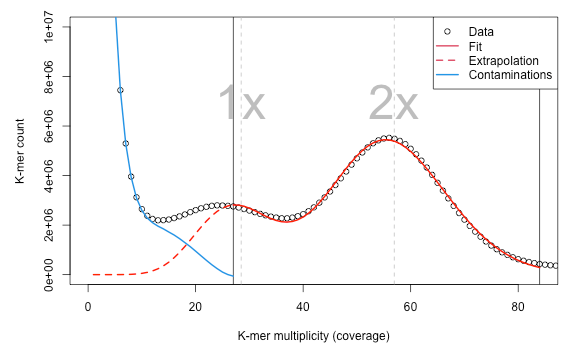 | DIPLOID MODEL, AUTO FITTED   k-mer length: 21   monoploid k-mer cov: 28.5   θ per k-mer: 0.1794   θ per nucleotide: 0.00854   non-rep GS (Mbp): 169.2   bias (peak width): 1  STARTING RANGES (MIN MAX)   monoploid k-mer cov: 10 44   log10 θ per k-mer: -2 0.6   non-rep GS (Mbp): 1 2000   bias (peak width): 0.1 3   x range: 27 84 |
| --- | --- |
| 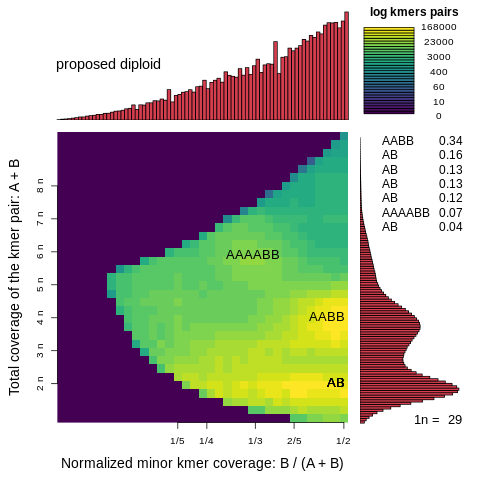 | 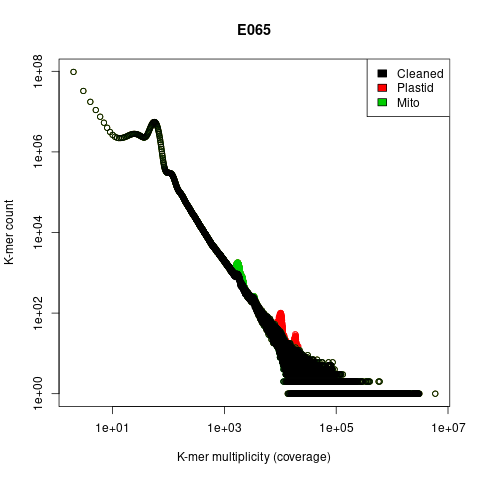 |

## Vi – *Euphrasia vigursii*

| 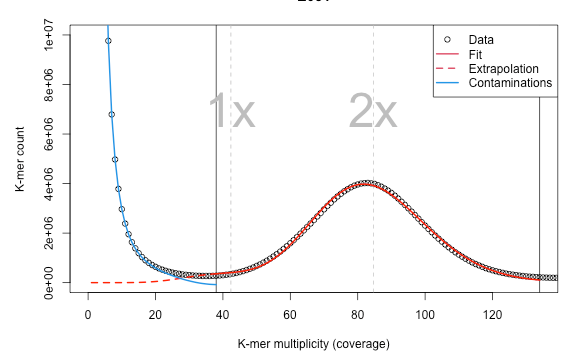 | DIPLOID MODEL, AUTO FITTED   k-mer length: 21   monoploid k-mer cov: 42.4   θ per k-mer: 0.0297   θ per nucleotide: 0.00142   non-rep GS (Mbp): 171.1   bias (peak width): 2.4  STARTING RANGES (MIN MAX)   monoploid k-mer cov: 10 63   log10 θ per k-mer: -2 0.6   non-rep GS (Mbp): 1 2000   bias (peak width): 0.1 3   x range: 38 134 |
| --- | --- |
| 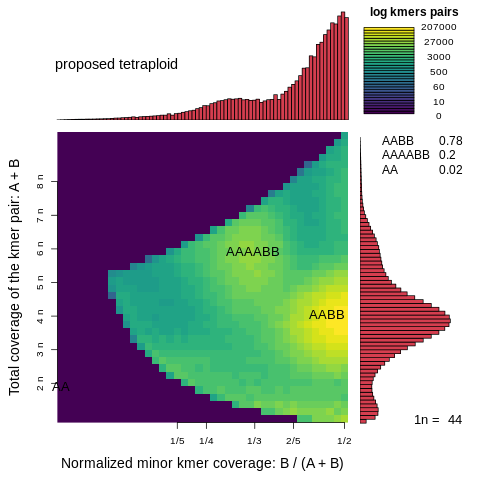 Smudgeplot wrongly proposes tetraploidy. The sample is actually diploid but with very low heterozygosity. The AABB peak corresponds to duplicated regions. | 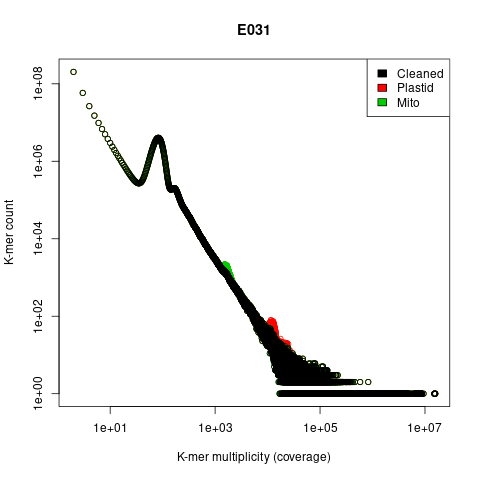 |

## Ro – *Euphrasia rostkoviana*

| 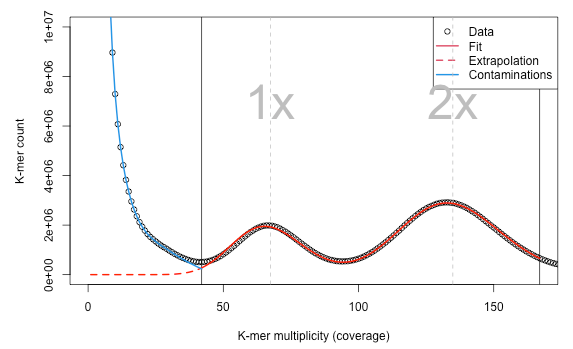 | DIPLOID MODEL, AUTO FITTED   k-mer length: 21   monoploid k-mer cov: 67.4   θ per k-mer: 0.2373   θ per nucleotide: 0.0113   non-rep GS (Mbp): 167.8   bias (peak width): 1.6  STARTING RANGES (MIN MAX)   monoploid k-mer cov: 10 100   log10 θ per k-mer: -2 0.6   non-rep GS (Mbp): 1 2000   bias (peak width): 0.1 3   x range: 42 167 |
| --- | --- |
| 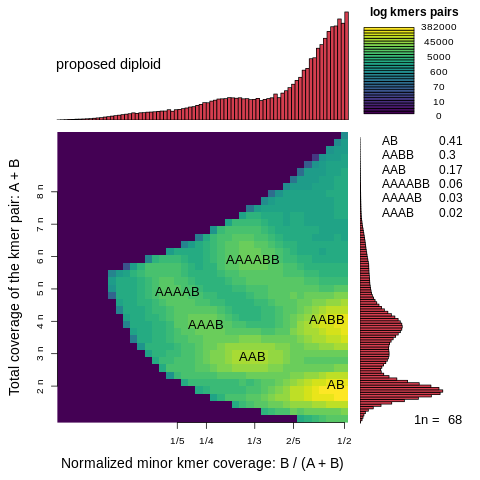 | 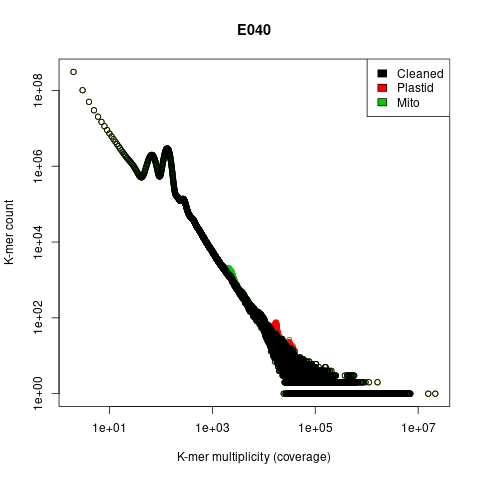 |

## Ri1 – *Euphrasia rivularis*

| 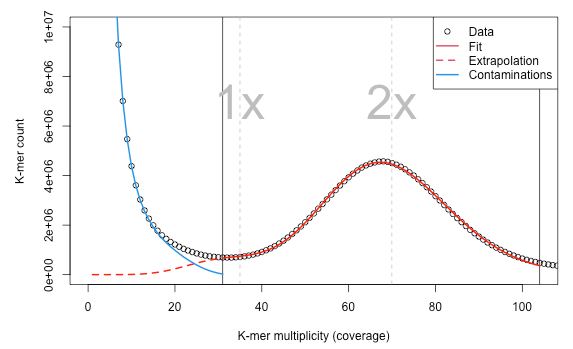 | DIPLOID MODEL, AUTO FITTED   k-mer length: 21   monoploid k-mer cov: 35   θ per k-mer: 0.049   θ per nucleotide: 0.00234   non-rep GS (Mbp): 170.9   bias (peak width): 2  STARTING RANGES (MIN MAX)   monoploid k-mer cov: 10 36   log10 θ per k-mer: -2 0.6   non-rep GS (Mbp): 1 2000   bias (peak width): 0.1 3   x range: 31 104 |
| --- | --- |
| 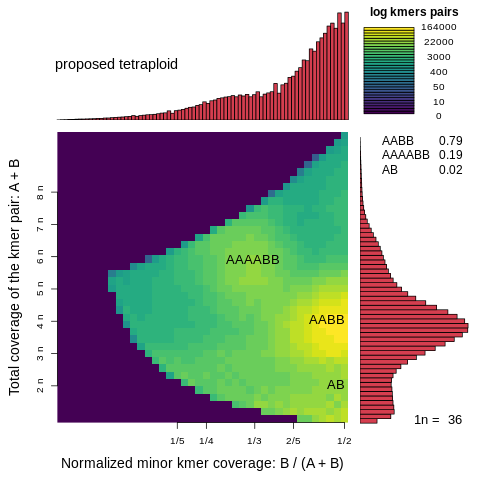 Smudgeplot wrongly proposes tetraploidy. The sample is actually diploid but with very low heterozygosity. The AABB peak corresponds to duplicated regions. | 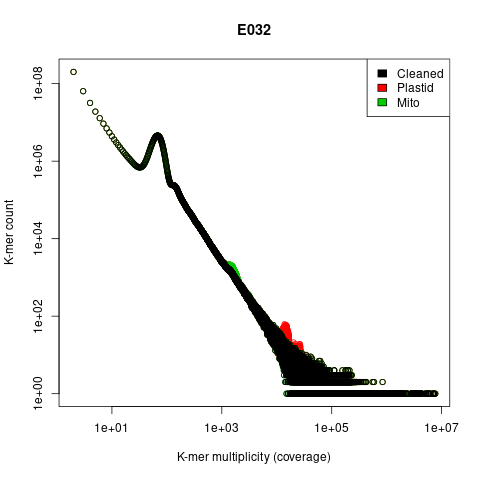 |

## Ri2 – *Euphrasia rivularis*

| 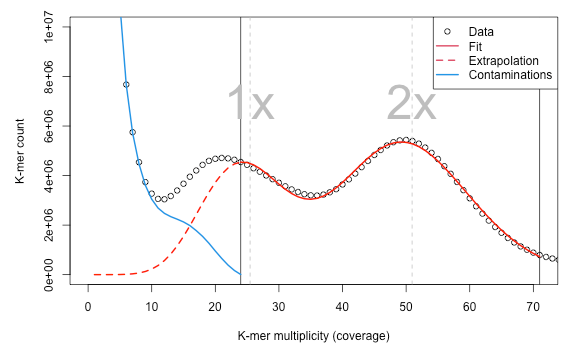 | DIPLOID MODEL, AUTO FITTED   k-mer length: 21   monoploid k-mer cov: 25.5   θ per k-mer: 0.2964   θ per nucleotide: 0.01411   non-rep GS (Mbp): 169.4   bias (peak width): 0.9  STARTING RANGES (MIN MAX)   monoploid k-mer cov: 10 41   log10 θ per k-mer: -2 0.6   non-rep GS (Mbp): 1 2000   bias (peak width): 0.1 3   x range: 24 71 |
| --- | --- |
| 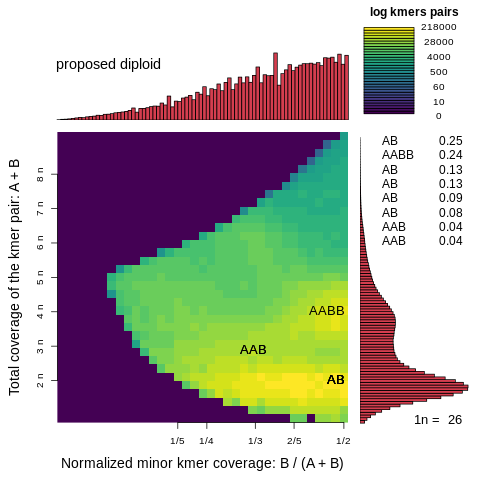 | 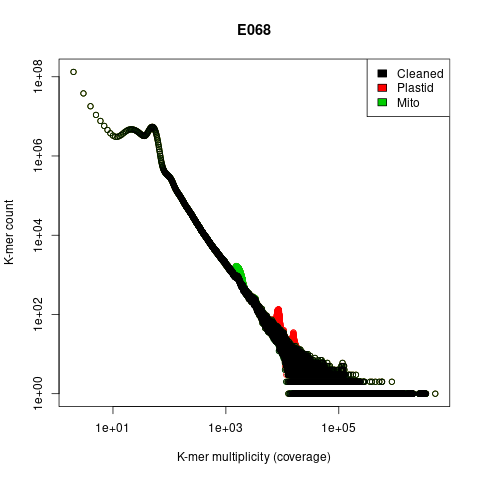 |

## Ri3 – *Euphrasia rivularis*

| 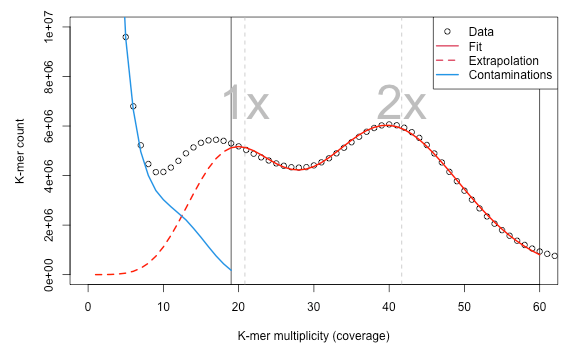 | DIPLOID MODEL, AUTO FITTED   k-mer length: 21   monoploid k-mer cov: 20.8   θ per k-mer: 0.297   θ per nucleotide: 0.01414   non-rep GS (Mbp): 171.5   bias (peak width): 1  STARTING RANGES (MIN MAX)   monoploid k-mer cov: 10 31   log10 θ per k-mer: -2 0.6   non-rep GS (Mbp): 1 2000   bias (peak width): 0.1 3   x range: 19 60 |
| --- | --- |
| 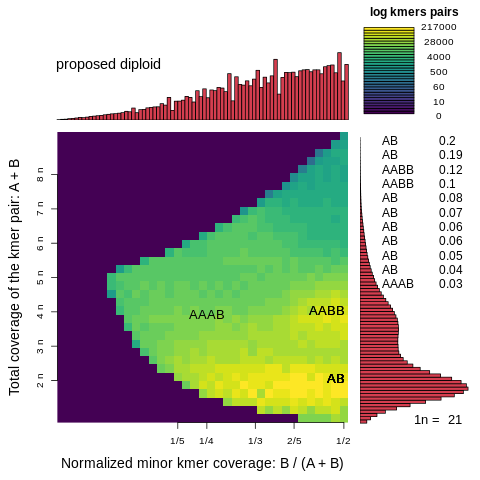 | 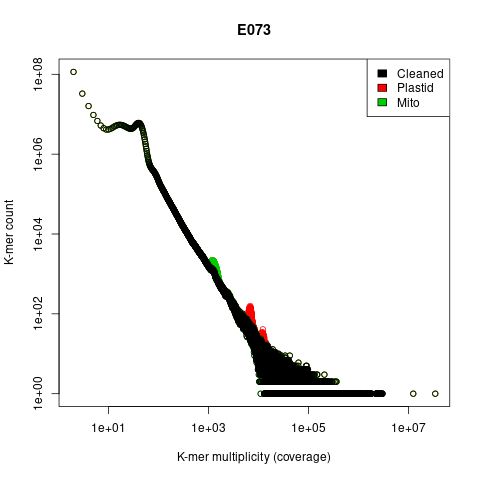 |

**
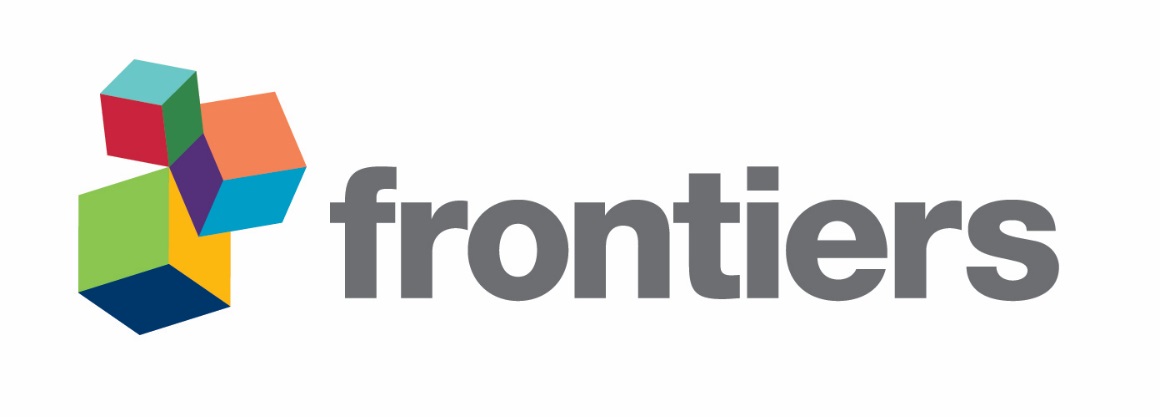
**
